# Supplementary figures and images for: Characterising the gut microbiome of stranded harbour seals (Phoca vitulina) in rehabilitation
Source: PLoS One. 2023 Dec 5;18(12):e0295072. doi: 10.1371/journal.pone.0295072 (PMC10697512; doi:10.1371/journal.pone.0295072)

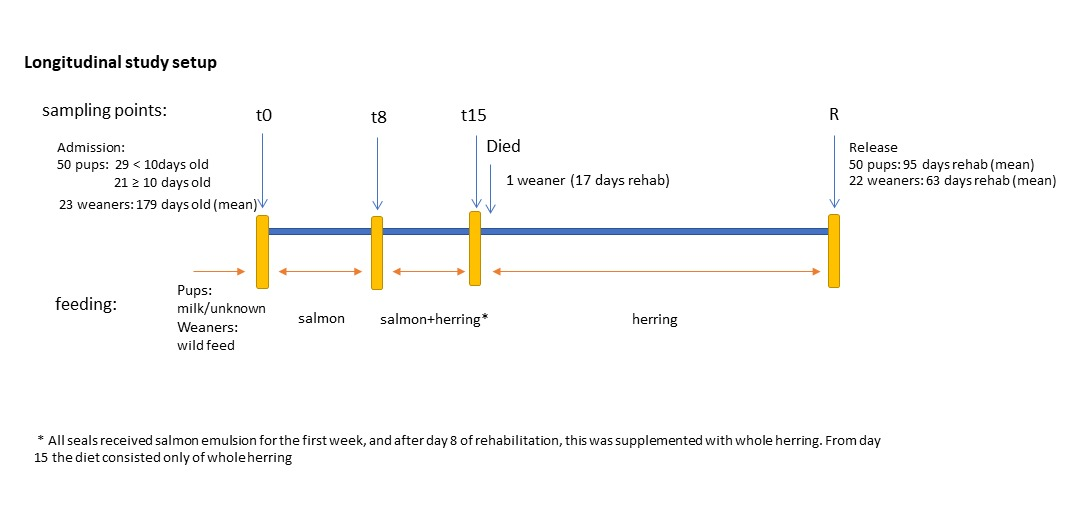

Supplement: S1 Fig — (TIF) [file pone.0295072.s001.tif]

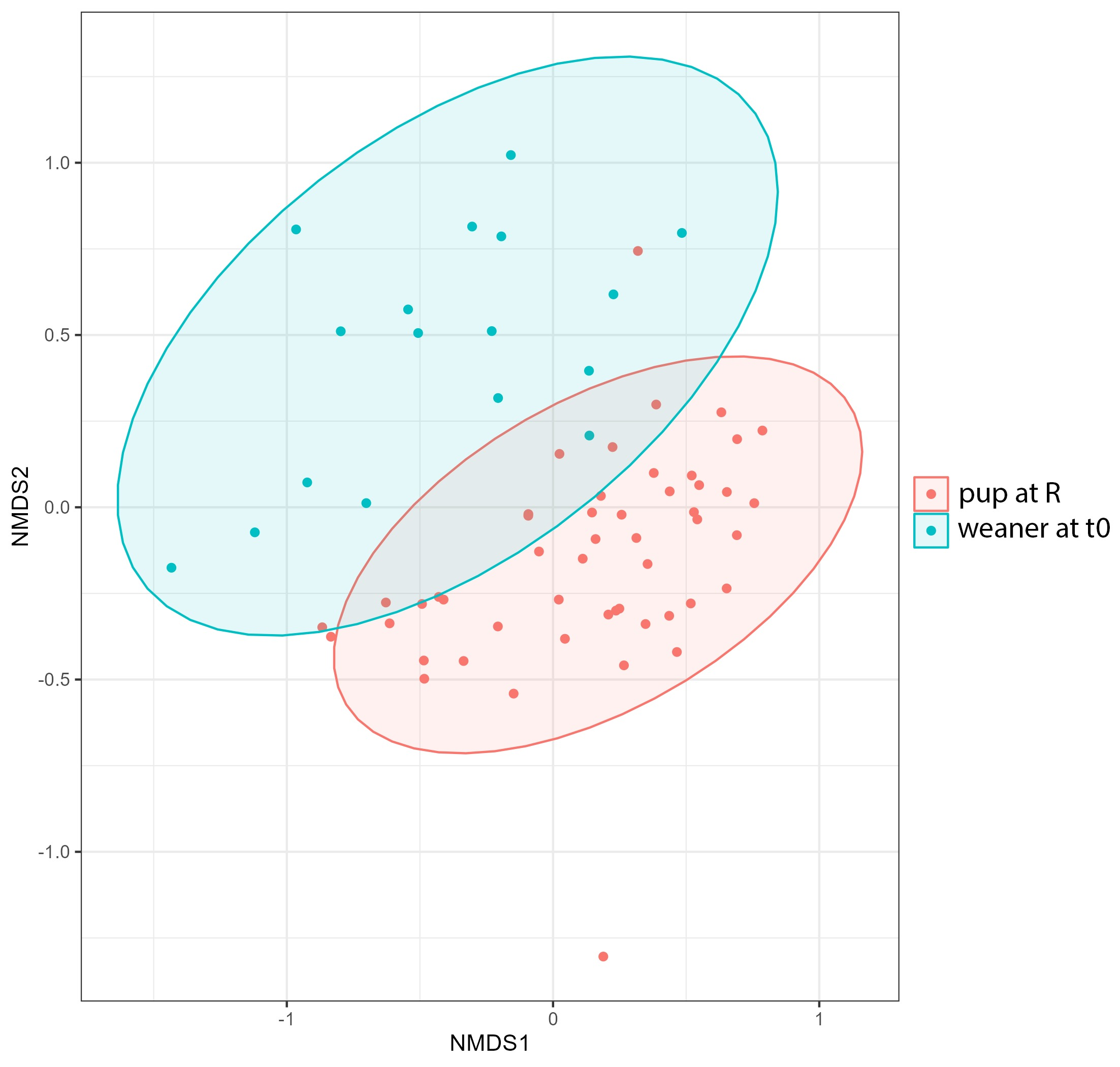

Supplement: S2 Fig — NMDS plots of the composition differences between two groups: 17 weaners at t0 and 50 pups at R of similar age (between 75 and 135 days old). Coloured dots and ellipses match the two groups. PERMANOVA p-value<0.001, R2 = 0.091, and ß dispersion’s p-value = 0.002. Stress value (2D) is 0.16. (TIF) [file pone.0295072.s002.tif]
