# Supplementary material for: Characterising the gut microbiome of stranded harbour seals (Phoca vitulina) in rehabilitation
Source: PLoS One. 2023 Dec 5;18(12):e0295072. doi: 10.1371/journal.pone.0295072 (PMC10697512; doi:10.1371/journal.pone.0295072)
Supplement: S1 Table — Significance code 0 ‘***’, 0.001 ‘**’, 0.01 ‘*’. (DOCX) [file pone.0295072.s003.docx]

S1 Table. Results of multivariable analysis (PERMANOVA) of the microbiome composition (beta diversity) at admission (t0) of pups.

|  | t0 | |
| --- | --- | --- |
|  | p-value | R^2^ |
| Age (days) | 0.003*** | 0.04 |
| Sex (ref=female) | 0.064 | 0.03 |
| Initial weight (Kg during admission) | 0.001*** | 0.05 |

Significance level codes: 0 ‘***’, 0.001 ‘**’, 0.01 ‘*’.
