# Supplementary material for: Characterising the gut microbiome of stranded harbour seals (Phoca vitulina) in rehabilitation
Source: PLoS One. 2023 Dec 5;18(12):e0295072. doi: 10.1371/journal.pone.0295072 (PMC10697512; doi:10.1371/journal.pone.0295072)
Supplement: S2 Table — a. Results of univariable analysis (Univariable Linear Mixed Effect Model) of the microbiome richness (observed index) of pups across time in rehabilitation. Significance code 0 ‘***’, 0.001 ‘**’, 0.01 ‘*’. b. Results of univariable analysis (Univariable Linear Mixed Effect Model) of the microbiome Shannon index of pups across time in rehabilitation. Significance code 0 ‘***’, 0.001 ‘**’, 0.01 ‘*’. c. Results of univariable analysis (Univariable Linear Mixed Effect Model) of the microbiome richness (observed index) of weaners across time in rehabilitation. Significance code 0 ‘***’, 0.001 ‘**’, 0.01 ‘*’. d. Results of univariable analysis (Univariable Linear Mixed Effect Model) of the microbiome Shannon index of weaners across time in rehabilitation. Significance code 0 ‘***’, 0.001 ‘**’, 0.01 ‘*’. (ZIP) [file pone.0295072.s004.zip › S2a_Table.docx]

S2.a Table. Results of univariable analysis (Univariable Linear Mixed Effect Model) of the microbiome richness (observed index) of pups across time in rehabilitation.

|  | group | p-value | estimate |
| --- | --- | --- | --- |
| Age (days) |  | <0.001*** | 0.25 |
| Feed | milk | 0.306 | -8.02 |
|  | salmon | 0.002** | -20.04 |
|  | unknown | 0.186 | -11.84 |
| Sex (ref=female) |  | 0.295 | 0.25 |
| Length of stay (days) |  | <0.001*** | 0.25 |

Significance level codes: 0 ‘***’, 0.001 ‘**’, 0.01 ‘*’.
