# Supplementary material for: Characterising the gut microbiome of stranded harbour seals (Phoca vitulina) in rehabilitation
Source: PLoS One. 2023 Dec 5;18(12):e0295072. doi: 10.1371/journal.pone.0295072 (PMC10697512; doi:10.1371/journal.pone.0295072)
Supplement: S3 Table — Significance code 0 ‘***’, 0.001 ‘**’, 0.01 ‘*’. (DOCX) [file pone.0295072.s005.docx]

S3 Table. PERMANOVA results of beta diversity between each timepoint in pups and weaners.

|  | pup | | weaner | |
| --- | --- | --- | --- | --- |
| Paired timepoints | p-value | R^2^ | p-value | R^2^ |
| t0 VS t8 | 0.001*** | 0.06 | 0.0001*** | 0.11 |
| t8 VS t15 | 0.002** | 0.03 | 0.06 | 0.03 |
| t15 VS R | 0.001*** | 0.08 | 0.003** | 0.05 |
| t0 VS t15 | 0.001*** | 0.05 | 0.001*** | 0.11 |
| t0 VS R | 0.001*** | 0.13 | 0.001*** | 0.10 |
| t0 VS R | 0.001*** | 0.14 | 0.001*** | 0.08 |

Significance level codes: 0 ‘***’, 0.001 ‘**’.
