# Supplementary material for: Characterising the gut microbiome of stranded harbour seals (Phoca vitulina) in rehabilitation
Source: PLoS One. 2023 Dec 5;18(12):e0295072. doi: 10.1371/journal.pone.0295072 (PMC10697512; doi:10.1371/journal.pone.0295072)
Supplement: S1 File — (DOCX) [file pone.0295072.s008.docx]

---

title: "R Notebook"

output: html_notebook

---

The following script can be used to create all results (tables and plots) of the manuscript:

*Characterising the gut microbiome of stranded harbour seals (Phoca vitulina) in rehabilitation.*

Ana Rubio-Garcia1+, Aldert L. Zomer , Ruoshui Guo, John W.A. Rossen, Jan H. van Zeijl, Jaap A. Wagenaar, and Roosmarijn E.C. Luiken

+Author for correspondence (a.rubiogarcia@uu.nl).

Present address: Division of Infectious Diseases and Immunology, Utrecht University Faculty of Veterinary Medicine, Yalelaan 1, 3584 CL, the Netherlands

ABSTRACT

Animal rehabilitation centres provide a unique opportunity to study the microbiome of wild animals because subjects will be handled for their treatment and can therefore be sampled longitudinally. However, rehabilitation may have unintended consequences on the animals' microbiome because of a less varied and suboptimal diet, possible medical treatment and exposure to a different environment and human handlers. Our study describes the gut microbiome of two large seal cohorts, 50 pups (0-30 days old at arrival) and 23 weaners (more than 60 days old at arrival) of stranded harbour seals admitted for rehabilitation at the Sealcentre Pieterburen in the Netherlands, and the effect of rehabilitation on it. Faecal samples were collected from all seals at arrival, two times during rehabilitation and before release. Only seals that did not receive antimicrobial treatment were included in the study. The average time in rehabilitation was 95 days for the pups and 63 days for the weaners. We observed that during rehabilitation, there was an increase in the relative abundance of some of the Campylobacterota spp and Actinobacteriota spp. The alpha diversity of the pups’ microbiome increased significantly during their rehabilitation (p-value <0.05), while there were no significant changes in alpha diversity over time for weaners. We hypothesize that aging is the main reason for the observed changes in the pups’ microbiome. At release, the sex of a seal pup was significantly associated with the microbiome’s alpha (i.e., Shannon diversity was higher for male pups, p-value <0.001) and beta diversity (p-value 0.001). For weaners, variation in the microbiome composition (beta diversity) at release was partly explained by sex and age of the seal (p-values 0.002 and 0.003 respectively). We mainly observed variables known to change the gut microbiome composition (e.g., age and sex) and conclude that rehabilitation in itself had only minor effects on the gut microbiome of seal pups and seal weaners.

libraries

```{r}

library(phyloseq)

library(microbiome)

library(ggplot2)

library(DESeq2)

library(tidyverse)

library(ggpubr)

library(readxl)

library(vegan)

```

TABLE 1

```{r}

metadata_seals_new <- read_excel("metadata_in_sealcentre_March_2021.xlsx")

metadata_unt <- metadata_seals_new %>% filter(was_the_seal_treated_at_some_point == "no")

T0 <- metadata_unt %>%

filter(sampletype_just_for_indication == "t0") %>%

group_by(pup_or_weaner) %>%

summarise(

count = n(),

mean_age = mean(age_at_sampling),

age_10 = quantile(age_at_sampling, 0.10),

age_90 = quantile(age_at_sampling, 0.90),

age_sd = sd(age_at_sampling),

mean_weight = mean(initial_weight_kg),

weight_10 = quantile(initial_weight_kg, 0.10),

weight_90 = quantile(initial_weight_kg, 0.90),

weight_sd = sd(initial_weight_kg)

)

release <- metadata_unt %>%

filter(sampletype_just_for_indication == "R") %>%

group_by(pup_or_weaner) %>%

summarise(

count = n(),

mean_age = mean(age_at_sampling),

age_10 = quantile(age_at_sampling, 0.10),

age_90 = quantile(age_at_sampling, 0.90),

age_sd = sd(age_at_sampling),

mean_days = mean(days_at_rehab),

days_10 = quantile(days_at_rehab, 0.10),

days_90 = quantile(days_at_rehab, 0.90),

days_sd = sd(days_at_rehab)

)

```

General dataprep of phyloseq object for compositional analyses and beta diversity

```{r}

load("ps.sealcentre.RData")

ps_sealcentre.new

ps_un_sealcentre <- prune_samples(sample_data(ps_sealcentre.new)$was_the_seal_treated_at_some_point != "yes", ps_sealcentre.new) # untreated samples in sealcentre

ps_un_sealcentre

# Add New Column (the total number of days in the sealcentre at release) - rehab_days

rehab_days_newcol <- data.frame(sample_data(ps_un_sealcentre)) %>%

select("sampletype_just_for_indication", "seal", "days_at_rehab")

tb_tmp <- rehab_days_newcol %>% filter(sampletype_just_for_indication %in% c("R", "D"))

idx <- match(rehab_days_newcol$seal, tb_tmp$seal)

rehab_days_newcol %>% mutate(rehab_days = tb_tmp$days_at_rehab[idx])

rehab_days_newcol[c("16-176-0", "16-176-15", "16-176-8"), "rehab_days"] <- 15

rehab_days_newcol <- sample_data(rehab_days_newcol)

ps_un_sealcentre <- merge_phyloseq(ps_un_sealcentre, rehab_days_newcol)

# Add New Column (distinguish the herring at t8, t15 and R) - feed_group

feed_group_newcol <- data.frame(sample_data(ps_un_sealcentre)) %>%

mutate(FeedGroup = case_when(feed == "milk" ~ "milk",

feed == "unknown" ~ "unknown",

feed == "wildfeed" ~ "wildfeed",

feed == "salmon" ~ "salmon",

sampletype_just_for_indication == "t8" & feed == "herring" ~ "herring_t8",

sampletype_just_for_indication == "t15" & feed == "herring" ~ "herring_t15",

sampletype_just_for_indication == "R" & feed == "herring" ~ "herring_R"))

rownames(feed_group_newcol) <- sample_names(ps_un_sealcentre)

feed_group_newcol <- sample_data(feed_group_newcol)

ps_un_sealcentre <- merge_phyloseq(ps_un_sealcentre, feed_group_newcol)

# Add new age column, dichotomous

age_newcol <- data.frame(sample_data(ps_un_sealcentre)) %>%

mutate(age_t0_cat = case_when(age_t0 < 10 ~ 0,

age_t0 == 10 ~ 1,

age_t0 > 10 ~ age_t0))

df<- age_newcol %>% filter(sampletype_just_for_indication == 't0')

table(df$pup_or_weaner, df$age_t0_cat)

age_newcol <- sample_data(age_newcol)

ps_un_sealcentre <- merge_phyloseq(ps_un_sealcentre, age_newcol)

### separate pups and weaners

ps_un_p_sealcentre <- prune_samples(sample_data(ps_un_sealcentre)$pup_or_weaner == "pup", ps_un_sealcentre)

ps_un_p_sealcentre %>% sample_data() %>% head()

ps_un_w_sealcentre <- prune_samples(sample_data(ps_un_sealcentre)$pup_or_weaner == "weaner", ps_un_sealcentre)

ps_un_w_sealcentre %>% sample_data() %>% head()

### calculate relative abundances

# the whole untreated group

ps_un_sealcentre.c <- microbiome::transform(ps_un_sealcentre, "compositional")

# pups

ps_un_p_sealcentre.c <- microbiome::transform(ps_un_p_sealcentre, "compositional")

# weaners

ps_un_w_sealcentre.c <- microbiome::transform(ps_un_w_sealcentre, "compositional")

```

FIGURE 1

```{r}

### FIGURE 1A

# Phylum level - top 6 species

pseq_phylum.un.p <- aggregate_rare(ps_un_p_sealcentre.c, level = "Phylum", detection = 4/100, prevalence = 5/100)

plot_composition(pseq_phylum.un.p,

otu.sort = "abundance",

average_by = "sampletype_just_for_indication") +

scale_x_discrete(limits = c("t0", "t8", "t15", "R")) +

labs(x = NULL,

y = "relative abundance",

title = "pup") +

scale_fill_discrete (name = "phylum") +

theme_bw() +

theme(text = element_text(size = 15))

### FIGURE 1B

ps_un_w.0d_sealcentre.c <- microbiome::remove_samples("15-291-17", ps_un_w_sealcentre.c) # remove timepoint D, sample ID is 15-291-17

# phylum level - top 6 species (report)

pseq_phylum.un.w <- aggregate_rare(ps_un_w.0d_sealcentre.c, level = "Phylum", detection = 0.1/100, prevalence = 50/100)

plot_composition(pseq_phylum.un.w,

otu.sort = "abundance",

average_by = "sampletype_just_for_indication") +

scale_x_discrete(limits = c("t0", "t8", "t15", "R")) +

labs(x = NULL,

y = "relative abundance",

title = "weaner") +

scale_fill_discrete (name = "phylum") +

theme_bw() +

theme(text = element_text(size = 15))

```

General data prep for TABLE 2 AND 3

```{r}

# pup

# Observed

obs_r.un.p <- estimate_richness(ps_r_un_p, split = TRUE, measures = "Observed")

rownames(obs_r.un.p) <- sample_names(ps_r_un_p)

obs_r.un.p <- sample_data(obs_r.un.p)

ps_r_un_p_obs <- merge_phyloseq(ps_r_un_p, obs_r.un.p)

df.r.un.p.obs <- data.frame(sample_data(ps_r_un_p_obs))

df.r.un.p.obs <- within(df.r.un.p.obs,

sampletype_just_for_indication <- factor(sampletype_just_for_indication, levels = c("t0", "t8", "t15", "R")))

# Shannon

shannon_r.un.p <- estimate_richness(ps_r_un_p, split = TRUE, measures = "Shannon")

rownames(shannon_r.un.p) <- sample_names(ps_r_un_p)

shannon_r.un.p <- sample_data(shannon_r.un.p)

ps_r_un_p_shannon <- merge_phyloseq(ps_r_un_p, shannon_r.un.p)

df.r.un.p.shannon <- data.frame(sample_data(ps_r_un_p_shannon))

df.r.un.p.shannon <- within(df.r.un.p.shannon,

sampletype_just_for_indication <- factor(sampletype_just_for_indication, levels = c("t0", "t8", "t15", "R")))

# weaner

# Observed

obs_r.un.w <- estimate_richness(ps_r_un_w, split = TRUE, measures = "Observed")

rownames(obs_r.un.w) <- sample_names(ps_r_un_w)

obs_r.un.w <- sample_data(obs_r.un.w)

ps_r_un_w_obs <- merge_phyloseq(ps_r_un_w, obs_r.un.w)

df.r.un.w.obs <- data.frame(sample_data(ps_r_un_w_obs)) %>%

filter(sampletype_just_for_indication != "D")

df.r.un.w.obs <- within(df.r.un.w.obs,

sampletype_just_for_indication <- factor(sampletype_just_for_indication, levels = c("t0", "t8", "t15", "R"))) # set the level

# Shannon

shannon_r.un.w <- estimate_richness(ps_r_un_w, split = TRUE, measures = "Shannon")

rownames(shannon_r.un.w) <- sample_names(ps_r_un_w)

shannon_r.un.w <- sample_data(shannon_r.un.w)

ps_r_un_w_shannon <- merge_phyloseq(ps_r_un_w, shannon_r.un.w)

df.r.un.w.shannon <- data.frame(sample_data(ps_r_un_w_shannon)) %>%

filter(sampletype_just_for_indication != "D")

df.r.un.w.shannon <- within(df.r.un.w.shannon,

sampletype_just_for_indication <- factor(sampletype_just_for_indication, levels = c("t0", "t8", "t15", "R"))) # set the level

# pups:

df.r.un.p.alpha.div <- merge(df.r.un.p.obs, df.r.un.p.shannon)

# weaners:

df.r.un.w.alpha.div <- merge(df.r.un.w.obs, df.r.un.w.shannon)

# t0

# pups:

df.r.un.p.alpha.div_t0 <- df.r.un.p.alpha.div %>% filter(sampletype_just_for_indication == "t0")

# df.r.un.p.alpha.div_t0 <- within(df.r.un.p.alpha.div_t0, feed = factor(feed, levels()))

# weaners:

df.r.un.w.alpha.div_t0 <- df.r.un.w.alpha.div %>% filter(sampletype_just_for_indication == "t0")

# R

# pups:

df.r.un.p.alpha.div_R <- df.r.un.p.alpha.div %>% filter(sampletype_just_for_indication == "R")

# weaners:

df.r.un.w.alpha.div_R <- df.r.un.w.alpha.div %>% filter(sampletype_just_for_indication == "R")

```

TABLE 2 AND 3

```{r}

# pups t0

# Observed

lm.p.obs.t0.age <- lm(Observed ~ age_t0_cat, data = df.r.un.p.alpha.div_t0)

summary(lm.p.obs.t0.age)

confint(lm.p.obs.t0.age, "age_t0_cat", level = 0.95)

lm.p.obs.t0.sex <- lm(Observed ~ sex, data = df.r.un.p.alpha.div_t0)

summary(lm.p.obs.t0.sex)

lm.p.obs.t0.weight <- lm(Observed ~ initial_weight_kg, data = df.r.un.p.alpha.div_t0)

summary(lm.p.obs.t0.weight)

confint(lm.p.obs.t0.weight, "initial_weight_kg", level = 0.95)

# Shannon

lm.p.shannon.t0.age <- lm(Shannon ~ age_t0_cat, data = df.r.un.p.alpha.div_t0)

summary(lm.p.shannon.t0.age)

confint(lm.p.shannon.t0.age, "age_t0_cat", level = 0.95)

lm.p.shannon.t0.sex <- lm(Shannon ~ sex, data = df.r.un.p.alpha.div_t0)

summary(lm.p.shannon.t0.sex)

lm.p.shannon.t0.weight <- lm(Shannon ~ initial_weight_kg, data = df.r.un.p.alpha.div_t0)

summary(lm.p.shannon.t0.weight)

confint(lm.p.shannon.t0.weight, "initial_weight_kg", level = 0.95)

# weaners t0

# Observed

lm.w.obs.t0.age <- lm(Observed ~ age_at_sampling, data = df.r.un.w.alpha.div_t0)

summary(lm.w.obs.t0.age)

lm.w.obs.t0.sex <- lm(Observed ~ sex, data = df.r.un.w.alpha.div_t0)

summary(lm.w.obs.t0.sex)

lm.w.obs.t0.weight <- lm(Observed ~ initial_weight_kg, data = df.r.un.w.alpha.div_t0)

summary(lm.w.obs.t0.weight)

# Shannon

lm.w.shannon.t0.age <- lm(Shannon ~ age_at_sampling, data = df.r.un.w.alpha.div_t0)

summary(lm.w.shannon.t0.age)

lm.w.shannon.t0.sex <- lm(Shannon ~ sex, data = df.r.un.w.alpha.div_t0)

summary(lm.w.shannon.t0.sex)

lm.w.shannon.t0.weight <- lm(Shannon ~ initial_weight_kg, data = df.r.un.w.alpha.div_t0)

summary(lm.w.shannon.t0.weight)

# PUPS R

# Observed

lm.p.obs.R.day <- lm(Observed ~ days_at_rehab, data = df.r.un.p.alpha.div_R)

summary(lm.p.obs.R.day)

lm.p.obs.R.sex <- lm(Observed ~ sex, data = df.r.un.p.alpha.div_R)

summary(lm.p.obs.R.sex)

# Shannon

lm.p.shannon.R.day <- lm(Shannon ~ days_at_rehab, data = df.r.un.p.alpha.div_R)

summary(lm.p.shannon.R.day)

lm.p.shannon.R.sex <- lm(Shannon ~ sex, data = df.r.un.p.alpha.div_R)

summary(lm.p.shannon.R.sex)

WEANERS

# Observed

lm.w.obs.R.age <- lm(Observed ~ age_at_sampling, data = df.r.un.w.alpha.div_R)

summary(lm.w.obs.R.age)

lm.w.obs.R.day <- lm(Observed ~ days_at_rehab, data = df.r.un.w.alpha.div_R)

summary(lm.w.obs.R.day)

lm.w.obs.R.sex <- lm(Observed ~ sex, data = df.r.un.w.alpha.div_R)

summary(lm.w.obs.R.sex)

# Shannon

lm.w.shannon.R.age <- lm(Shannon ~ age_at_sampling, data = df.r.un.w.alpha.div_R)

summary(lm.w.shannon.R.age)

lm.w.shannon.R.day <- lm(Shannon ~ days_at_rehab, data = df.r.un.w.alpha.div_R)

summary(lm.w.shannon.R.day)

lm.w.shannon.R.sex <- lm(Shannon ~ sex, data = df.r.un.w.alpha.div_R)

summary(lm.w.shannon.R.sex)

#check assumptions and correlations (if needed)

qqnorm()

shapiro.test()

chisq.test()

cor.test(x,y, method = "spearman")

```

TABLE 4 AND 5 AND SUPPLEMENTAL TABLE 1

```{r}

# t0

# pups

ps_un_p_sealcentre.t0.c <- prune_samples(sample_data(ps_un_p_sealcentre.c)$sampletype_just_for_indication == "t0", ps_un_p_sealcentre.c)

# weaners

ps_un_w_sealcentre.t0.c <- prune_samples(sample_data(ps_un_w_sealcentre.c)$sampletype_just_for_indication == "t0", ps_un_w_sealcentre.c)

# pups in t0

ps_un_p_sealcentre.t0.bray <- phyloseq::distance(ps_un_p_sealcentre.t0.c, method = "bray")

df.ps_un_p_sealcentre.t0 <- data.frame(sample_data(ps_un_p_sealcentre.t0.c))

# weaners in t0

ps_un_w_sealcentre.t0.bray <- phyloseq::distance(ps_un_w_sealcentre.t0.c, method = "bray")

df.ps_un_w_sealcentre.t0 <- data.frame(sample_data(ps_un_w_sealcentre.t0.c))

# pups

adonis2(ps_un_p_sealcentre.t0.bray ~ age_t0_cat, data = df.ps_un_p_sealcentre.t0, by = "margin")

adonis2(ps_un_p_sealcentre.t0.bray ~ sex, data = df.ps_un_p_sealcentre.t0, by = "margin")

mod.un.p.centre.t0.sex <- betadisper(ps_un_p_sealcentre.t0.bray, df.ps_un_p_sealcentre.t0$sex)

anova(mod.un.p.centre.t0.sex)

#check for correlation if needed

chisq.test()

cor.test(x,y, method = "spearman")

adonis2(ps_un_p_sealcentre.t0.bray ~ age_t0_cat + initial_weight_kg + sex, data = df.ps_un_p_sealcentre.t0, by = "margin")

# weaners

adonis2(ps_un_w_sealcentre.t0.bray ~ age_at_sampling, data = df.ps_un_w_sealcentre.t0, by = "margin")

adonis2(ps_un_w_sealcentre.t0.bray ~ sex, data = df.ps_un_w_sealcentre.t0, by = "margin")

mod.un.w.centre.t0.sex <- betadisper(ps_un_w_sealcentre.t0.bray, df.ps_un_w_sealcentre.t0$sex)

anova(mod.un.w.centre.t0.sex)

adonis2(ps_un_w_sealcentre.t0.bray ~ initial_weight_kg, data = df.ps_un_w_sealcentre.t0, by = "margin")

# R

# pups

ps_un_p_sealcentre.R.c <- prune_samples(sample_data(ps_un_p_sealcentre.c)$sampletype_just_for_indication == "R", ps_un_p_sealcentre.c)

# weaners

ps_un_w_sealcentre.R.c <- prune_samples(sample_data(ps_un_w_sealcentre.c)$sampletype_just_for_indication == "R", ps_un_w_sealcentre.c)

# pups in R

ps_un_p_sealcentre.R.bray <- phyloseq::distance(ps_un_p_sealcentre.R.c, method = "bray")

df.ps_un_p_sealcentre.R <- data.frame(sample_data(ps_un_p_sealcentre.R.c))

# weaners in R

ps_un_w_sealcentre.R.bray <- phyloseq::distance(ps_un_w_sealcentre.R.c, method = "bray")

df.ps_un_w_sealcentre.R <- data.frame(sample_data(ps_un_w_sealcentre.R.c))

# pups

adonis2(ps_un_p_sealcentre.R.bray ~ age_at_sampling, data = df.ps_un_p_sealcentre.R, by = "margin")

adonis2(ps_un_p_sealcentre.R.bray ~ sex, data = df.ps_un_p_sealcentre.R, by = "margin")

mod.un.p.centre.R.sex <- betadisper(ps_un_p_sealcentre.R.bray, df.ps_un_p_sealcentre.R$sex)

anova(mod.un.p.centre.R.sex)

# weaners

adonis2(ps_un_w_sealcentre.R.bray ~ age_at_sampling, data = df.ps_un_w_sealcentre.R, by = "margin")

adonis2(ps_un_w_sealcentre.R.bray ~ sex, data = df.ps_un_w_sealcentre.R, by = "margin")

mod.un.w.centre.R.sex <- betadisper(ps_un_w_sealcentre.R.bray, df.ps_un_w_sealcentre.R$sex)

anova(mod.un.w.centre.R.sex)

```

FIGURE 3 and 4

```{r}

ord.nmds.bray.un.p.centre <- ordinate(ps_un_p_sealcentre.c, method = "NMDS", distance = "bray", k = 3, autotransform = FALSE)

ps_un_p_sealcentre.c@sam_data$sampletype_just_for_indication <- factor(

ps_un_p_sealcentre.c@sam_data$sampletype_just_for_indication,

levels = c("t0", "t8", "t15", "R", "D")

)

### pups

plot_ordination(ps_un_p_sealcentre.c, ord.nmds.bray.un.p.centre,

color="sampletype_just_for_indication")+

stat_ellipse(geom = "polygon", aes(fill = sampletype_just_for_indication), alpha = 0.25)+

theme_bw()+

theme(legend.title = element_blank())+

ggtitle("pups")

plot_ordination(ps_un_p_sealcentre.c, ord.nmds.bray.un.p.centre,

color="sampletype_just_for_indication",

axes = c(2,3))+

stat_ellipse(geom = "polygon", aes(fill = sampletype_just_for_indication), alpha = 0.25)+

theme_bw()+

theme(legend.title = element_blank())+

ggtitle("pups")

plot_ordination(ps_un_p_sealcentre.c, ord.nmds.bray.un.p.centre,

color="sampletype_just_for_indication",

axes = c(1,3))+

stat_ellipse(geom = "polygon", aes(fill = sampletype_just_for_indication), alpha = 0.25)+

theme_bw()+

theme(legend.title = element_blank())+

ggtitle("pups")

### weaners

# remove dead one

ps_un_w_sealcentre.c_mind <-

prune_samples(sample_data(ps_un_w_sealcentre.c)$sampletype_just_for_indication != "D", ps_un_w_sealcentre.c)

ps_un_w_sealcentre.c

ps_un_w_sealcentre.c_mind

ord.nmds.bray.un.w.centre <- ordinate(ps_un_w_sealcentre.c_mind, method = "NMDS", distance = "bray", k = 3, autotransform = FALSE)

ps_un_w_sealcentre.c_mind@sam_data$sampletype_just_for_indication <- factor(

ps_un_w_sealcentre.c_mind@sam_data$sampletype_just_for_indication,

levels = c("t0", "t8", "t15", "R")

)

plot_ordination(ps_un_w_sealcentre.c_mind, ord.nmds.bray.un.w.centre,

color="sampletype_just_for_indication")+

stat_ellipse(geom = "polygon", aes(fill = sampletype_just_for_indication), alpha = 0.25)+

theme_bw()+

theme(legend.title = element_blank())+

ggtitle("weaners")

plot_ordination(ps_un_w_sealcentre.c_mind, ord.nmds.bray.un.w.centre,

color="sampletype_just_for_indication",

axes = c(2,3))+

stat_ellipse(geom = "polygon", aes(fill = sampletype_just_for_indication), alpha = 0.25)+

theme_bw()+

theme(legend.title = element_blank())+

ggtitle("weaners")

plot_ordination(ps_un_w_sealcentre.c_mind, ord.nmds.bray.un.w.centre,

color="sampletype_just_for_indication",

axes = c(1,3))+

stat_ellipse(geom = "polygon", aes(fill = sampletype_just_for_indication), alpha = 0.25)+

theme_bw()+

theme(legend.title = element_blank())+

ggtitle("weaners")

```

General data prep of phyloseq object of rarefied data for alpha diversity analyses

```{r}

# ps.rarefied <- rarefy_even_depth(ps)

# ps.rarefied

# save(ps.rarefied, file = "ps.rarefied.RData")

load("ps.rarefied.sealcentre.RData")

ps_r_sealcentre.new

ps_r_un_sealcentre <- prune_samples(sample_data(ps_r_sealcentre.new)$was_the_seal_treated_at_some_point != "yes", ps_r_sealcentre.new) # rarefied untreated samples in sealcentre

ps_r_un_sealcentre

# Add New Column (the total number of days in the sealcentre at release) - rehab_days

rehab_days_newcol <- data.frame(sample_data(ps_r_un_sealcentre)) %>%

select("sampletype_just_for_indication", "seal", "days_at_rehab")

tb_tmp <- rehab_days_newcol %>% filter(sampletype_just_for_indication %in% c("R", "D"))

idx <- match(rehab_days_newcol$seal, tb_tmp$seal)

rehab_days_newcol %<>% mutate(rehab_days = tb_tmp$days_at_rehab[idx])

rehab_days_newcol[c("16-176-0", "16-176-15", "16-176-8"), "rehab_days"] <- 15

rehab_days_newcol <- sample_data(rehab_days_newcol) # transfer to the sample_data()

ps_r_un_sealcentre <- merge_phyloseq(ps_r_un_sealcentre, rehab_days_newcol) # combine this new column with the phyloseq object

# Add New Column (distinguish the herring at t8, t15 and R) - feed_group

feed_group_newcol <- data.frame(sample_data(ps_r_un_sealcentre)) %>%

mutate(FeedGroup = case_when(feed == "milk" ~ "milk",

feed == "unknown" ~ "unknown",

feed == "wildfeed" ~ "wildfeed",

feed == "salmon" ~ "salmon",

sampletype_just_for_indication == "t8" & feed == "herring" ~ "herring_t8",

sampletype_just_for_indication == "t15" & feed == "herring" ~ "herring_t15",

sampletype_just_for_indication == "R" & feed == "herring" ~ "herring_R"))

rownames(feed_group_newcol) <- sample_names(ps_r_un_sealcentre)

feed_group_newcol <- sample_data(feed_group_newcol)

ps_r_un_sealcentre <- merge_phyloseq(ps_r_un_sealcentre, feed_group_newcol)

# Add new age column, dichotomous

age_newcol <- data.frame(sample_data(ps_r_un_sealcentre)) %>%

mutate(age_t0_cat = case_when(age_t0 < 10 ~ 0,

age_t0 == 10 ~ 1,

age_t0 > 10 ~ age_t0))

df<- age_newcol %>% filter(sampletype_just_for_indication == 't0')

table(df$pup_or_weaner, df$age_t0_cat)

age_newcol <- sample_data(age_newcol)

ps_r_un_sealcentre <- merge_phyloseq(ps_r_un_sealcentre, age_newcol)

ps_r_un_sealcentre_0d <-

prune_samples(sample_data(ps_r_un_sealcentre)$sampletype_just_for_indication != "D", ps_r_un_sealcentre) # rarefied untreated samples without one dead sample in sealcentre

ps_r_un_sealcentre_0d

# rarefied untreated pup grouped at the admission day

ps_r_un_p <- prune_samples(sample_data(ps_r_un_sealcentre)$pup_or_weaner == "pup", ps_r_un_sealcentre)

# rarefied untreated weaner grouped at the admission day

ps_r_un_w <- prune_samples(sample_data(ps_r_un_sealcentre)$pup_or_weaner == "weaner", ps_r_un_sealcentre)

```

FIGURE 2

```{r}

# alpha diversity metrics of rarefied untreated pups grouped at the admission day for T Test:

ps.r.un.p_m <- estimate_richness(ps_r_un_p, measures = c("Observed", "Chao1", "Shannon", "Simpson"))

df.r.un.p <- data.frame(sample_data(ps_r_un_p))

timepoint_id_p <- select(df.r.un.p, TimePoint = "sampletype_just_for_indication", ID = "seal", Feed = "feed")

ps.r.un.p_t <- cbind(ps.r.un.p_m, timepoint_id_p) %>%

arrange(TimePoint, ID) # data set for T-Test (including all time points)

ps.r.un.p_t <- within(ps.r.un.p_t, TimePoint <- factor(TimePoint, levels = c("t0", "t8", "t15", "R"))) # set the level for ggplot

ps.r.un.p_t <- within(ps.r.un.p_t, Feed <- factor(Feed, levels = c("milk", "unknown", "salmon", "herring"))) # set the level for ggplot

# alpha diversity metrics of rarefied untreated weaners grouped at the admission day for T Test:

ps.r.un.w_m.0 <- estimate_richness(ps_r_un_w, measures = c("Observed", "Chao1", "Shannon", "Simpson"))

df.r.un.w.0 <- data.frame(sample_data(ps_r_un_w))

timepoint_w.0 <- select(df.r.un.w.0, TimePoint = "sampletype_just_for_indication", ID = "seal", Feed = "feed")

ps.r.un.w_t.0 <- cbind(ps.r.un.w_m.0, timepoint_w.0) %>%

arrange(TimePoint, ID) # data set for T-Test (including all time points)

ps.r.un.w_t.0 <- within(ps.r.un.w_t.0, TimePoint <- factor(TimePoint, levels = c("t0", "t8", "t15", "R", "D"))) # set the level for ggplot

ps.r.un.w_t.0 <- within(ps.r.un.w_t.0, TimePoint <- factor(TimePoint, levels = c("t0", "t8", "t15", "R", "D"))) # set the level for ggplot

# PUP Observerd

ggplot(ps.r.un.p_t,

aes(x = TimePoint, y = Observed, fill = Feed)) +

geom_violin(trim = FALSE) +

geom_boxplot(alpha = 0.6, fill = "white") +

geom_signif(comparisons = list(c("t0", "t8"),

c("t8", "t15"),

c("t15", "R")),

test = "t.test",

test.args = list(paired = TRUE),

map_signif_level = TRUE,

y_position = c(345, 345, 345)) +

geom_signif(comparisons = list(c("t0", "t15"),

c("t0", "R"),

c("t8", "R")),

test = "t.test",

test.args = list(paired = TRUE),

map_signif_level = TRUE,

y_position = c(365, 385, 405)) +

labs(x = "Timepoint", y = "Observed Index") +

scale_fill_discrete(name = "Feed") +

theme_bw() +

theme_classic()

# PUP Shannon

ggplot(ps.r.un.p_t,

aes(x = TimePoint, y = Shannon, fill = Feed)) +

geom_violin(trim = FALSE) +

geom_boxplot(alpha = 0.6, fill = "white") +

geom_signif(comparisons = list(c("t0", "t8"),

c("t8", "t15"),

c("t15", "R")),

test = "t.test",

test.args = list(paired = TRUE),

map_signif_level = TRUE,

y_position = c(5.5, 5.5, 5.5)) +

geom_signif(comparisons = list(c("t0", "t15"),

c("t0", "R"),

c("t8", "R")),

test = "t.test",

test.args = list(paired = TRUE),

map_signif_level = TRUE,

y_position = c(5.75, 6, 6.25)) +

labs(x = "Timepoint", y = "Shannon Index") +

scale_fill_discrete(name = "Feed") +

theme_bw() +

theme_classic()

# weaners

ps.r.un.w_t <- ps.r.un.w_t.0 %>% filter(TimePoint != "D")

# WEANER Observed

ggplot(ps.r.un.w_t,

aes(x = TimePoint, y = Observed, fill = Feed)) +

geom_violin(trim = FALSE) +

geom_boxplot(alpha = 0.6, fill = "white") +

# ggtitle("The differences of Observed between each time-point (weaners)") +

scale_fill_discrete(name = "Feed") +

labs(x = "Timepoint", y = "Observed Index") +

theme_bw() +

theme_classic()

# WEANER Shannon

ggplot(ps.r.un.w_t,

aes(x = TimePoint, y = Shannon, fill = Feed)) +

geom_violin(trim = FALSE) +

geom_boxplot(alpha = 0.6, fill = "white") +

scale_fill_discrete(name = "Feed") +

labs(x = "Timepoint", y = "Shannon Index") +

theme_bw() +

theme_classic()

```

FIGURE 5 and figure S2 (age matched - pup release vs weaner arrival)

```{r}

### Analysis of alpha diversity

load("ps.rarefied.sealcentre.RData")

ps_r_sealcentre.new # rarefied object

ps_r_un.p.R_sealcentre <- prune_samples(sample_data(ps_r_sealcentre.new)$was_the_seal_treated_at_some_point != "yes" & sample_data(ps_r_sealcentre.new)$sampletype_just_for_indication == "R" & sample_data(ps_r_sealcentre.new)$pup_or_weaner == "pup", ps_r_sealcentre.new) # untreated pups at R in sealcentre

#data.frame(sample_data(ps_r_un.p.R_sealcentre)) %>% view()

ps_r_w.t0_sealcentre <- prune_samples(sample_data(ps_r_sealcentre.new)$sampletype_just_for_indication == "t0" & sample_data(ps_r_sealcentre.new)$pup_or_weaner == "weaner", ps_r_sealcentre.new) # all weaners at t0 in sealcentre

# merge two phyloseq objects

ps_r_age.match <- merge_phyloseq(ps_r_un.p.R_sealcentre, ps_r_w.t0_sealcentre)

# dataframe before selection

df.ps_r_age.match <- data.frame(sample_data(ps_r_age.match))

# selection of correct age group

ps_r_age.match.se <- prune_samples(sample_data(ps_r_age.match)$age_at_sampling < 135 & sample_data(ps_r_age.match)$age_at_sampling > 75, ps_r_age.match)

#create dataframe with alpha div.

# Observed

obs_r_age.match.se <- estimate_richness(ps_r_age.match.se, split = TRUE, measures = "Observed")

rownames(obs_r_age.match.se) <- sample_names(ps_r_age.match.se)

obs_r_age.match.se <- sample_data(obs_r_age.match.se)

ps_r_age.match.se_obs <- merge_phyloseq(ps_r_age.match.se, obs_r_age.match.se)

df.r.age.match.se.obs <- data.frame(sample_data(ps_r_age.match.se_obs))

# Chao1

chao1_r_age.match.se <- estimate_richness(ps_r_age.match.se, split = TRUE, measures = "Chao1")

rownames(chao1_r_age.match.se) <- sample_names(ps_r_age.match.se)

chao1_r_age.match.se <- sample_data(chao1_r_age.match.se)

ps_r_age.match.se_chao1 <- merge_phyloseq(ps_r_age.match.se, chao1_r_age.match.se)

df.r.age.match.se.chao1 <- data.frame(sample_data(ps_r_age.match.se_chao1)) # one more column is "se.chao1"

# Shannon

shannon_r_age.match.se <- estimate_richness(ps_r_age.match.se, split = TRUE, measures = "Shannon")

rownames(shannon_r_age.match.se) <- sample_names(ps_r_age.match.se)

shannon_r_age.match.se <- sample_data(shannon_r_age.match.se)

ps_r_age.match.se_shannon <- merge_phyloseq(ps_r_age.match.se, shannon_r_age.match.se)

df.r.age.match.se.shannon <- data.frame(sample_data(ps_r_age.match.se_shannon))

# Simpson

simpson_r_age.match.se <- estimate_richness(ps_r_age.match.se, split = TRUE, measures = "Simpson")

rownames(simpson_r_age.match.se) <- sample_names(ps_r_age.match.se)

simpson_r_age.match.se <- sample_data(simpson_r_age.match.se)

ps_r_age.match.se_simpson <- merge_phyloseq(ps_r_age.match.se, simpson_r_age.match.se)

df.r.age.match.se.simpson <- data.frame(sample_data(ps_r_age.match.se_simpson))

# final data-set with 4 alpha diversity metrics

df.r.am.se.alpha.div <- merge(df.r.age.match.se.obs, df.r.age.match.se.chao1) %>%

merge(df.r.age.match.se.shannon) %>%

merge(df.r.age.match.se.simpson)

# PLOT Observed

ggplot(df.r.am.se.alpha.div,

aes(x = pup_or_weaner, y = Observed, color = pup_or_weaner)) +

# geom_violin(trim = FALSE) +

geom_jitter() +

geom_boxplot(alpha = 0.6, fill = "white") +

geom_signif(comparisons = list(c("pup", "weaner")),

test = "wilcox.test",

test.args = list(paired = FALSE),

map_signif_level = TRUE, # p-value = 0.004

y_position = 235,

color = "black") +

labs(x = NULL, y = "Observed Index") +

scale_color_discrete(name = NULL, labels = c( "pup at R", "weaner at t0")) +

scale_x_discrete(limits = c("weaner", "pup"), labels = c("weaner at t0", "pup at R")) +

theme_classic()

# PLOT Shannon

ggplot(df.r.am.se.alpha.div,

aes(x = pup_or_weaner, y = Shannon, color = pup_or_weaner)) +

# geom_violin(trim = FALSE) +

geom_jitter() +

geom_boxplot(alpha = 0.6, fill = "white") +

geom_signif(comparisons = list(c("pup", "weaner")),

test = "wilcox.test",

test.args = list(paired = FALSE),

map_signif_level = TRUE, # p-value = 0.21

y_position = 4.8,

color = "black") +

labs(x = NULL, y = "Shannon Index") +

scale_color_discrete(name = NULL, labels = c("pup at R", "weaner at t0")) +

scale_x_discrete(limits = c("weaner", "pup"), labels = c("weaner at t0", "pup at R")) +

theme_classic()

### Analysis of beta diversity

load("ps.sealcentre.RData")

ps_sealcentre.new # standard un-rarefied object

#selection of correct samples

ps_un.p.R_sealcentre <- prune_samples(sample_data(ps_sealcentre.new)$was_the_seal_treated_at_some_point != "yes" & sample_data(ps_sealcentre.new)$sampletype_just_for_indication == "R" & sample_data(ps_sealcentre.new)$pup_or_weaner == "pup", ps_sealcentre.new) # untreated pups at R in sealcentre

data.frame(sample_data(ps_un.p.R_sealcentre)) %>% view()

ps_w.t0_sealcentre <- prune_samples(sample_data(ps_sealcentre.new)$sampletype_just_for_indication == "t0" & sample_data(ps_sealcentre.new)$pup_or_weaner == "weaner", ps_sealcentre.new) # all weaners at t0 in sealcentre

# merge two phyloseq objects

ps_age.match <- merge_phyloseq(ps_un.p.R_sealcentre, ps_w.t0_sealcentre)

# dataframe before selection

df.ps_age.match <- data.frame(sample_data(ps_age.match))

# selection of correct age group

ps_age.match.se <- prune_samples(sample_data(ps_age.match)$age_at_sampling < 135 & sample_data(ps_age.match)$age_at_sampling > 75, ps_age.match)

ps_age.match.se

ps_age.match.se.c <- microbiome::transform(ps_age.match.se, "compositional")

ps_age.match.se.bray <- phyloseq::distance(ps_age.match.se.c, method = "bray")

df.ps_age.match.se <- data.frame(sample_data(ps_age.match.se))

#PLOT nmds beta diversity

ord.nmds.bray.age.match <- ordinate(ps_age.match.se.c, method = "NMDS", distance = "bray", k = 3, autotransform = FALSE)

plot_ordination( ps_age.match.se.c, ord.nmds.bray.age.match,color="pup_or_weaner" )+

theme_bw()+

theme(legend.title = element_blank())+

#scale_color_manual(values = c("pup" = "hotpink1",

# "weaner"="coral4"))+

stat_ellipse(geom = "polygon", aes(fill = pup_or_weaner), alpha = 0.1)

ggsave("Age matched nmds supplemental figure 2.png")

#PERMANOVA of pup release vs weaner arrival

adonis2(ps_age.match.se.bray ~ pup_or_weaner, data = df.ps_age.match.se, by = "margin")

mod.age.match.se <- betadisper(ps_age.match.se.bray, df.ps_age.match.se$pup_or_weaner)

anova(mod.age.match.se)

```

FIGURE 6 (deseq)

```{r}

# removing rare species pups

ps.f_un_p_sealcentre <- filter_taxa(ps_un_p_sealcentre, function(x) sum(x > 3) > (0.2*length(x)), TRUE)

ps.f_un_p_sealcentre

ps.f_un_p_sealcentre.pseudo <- microbiome::transform(ps.f_un_p_sealcentre, 'shift', shift = 1)

dds.un.p.centre.pseudo.adj <- phyloseq_to_deseq2(ps.f_un_p_sealcentre.pseudo, ~ sex + sampletype_just_for_indication)

dds.un.p.centre.pseudo.adj

diagdds.un.p.centre.pseudo.adj <- DESeq(dds.un.p.centre.pseudo.adj, test = "Wald", fitType = "local")

diagdds.un.p.centre.pseudo.adj

resultsNames(diagdds.un.p.centre.pseudo.adj) #

summary(diagdds.un.p.centre.pseudo.adj) #

plotDispEsts(diagdds.un.p.centre.pseudo.adj)

# final object

res.un.p.centre.pseudo.adj <- results(diagdds.un.p.centre.pseudo.adj, contrast = c("sampletype_just_for_indication", "R", "t0"))

res.un.p.centre.pseudo.adj %>% head()

res.un.p.centre.pseudo.adj %>% summary()

alpha <- 0.01

sigtab.un.p.centre.pseudo.adj <- res.un.p.centre.pseudo.adj[which(res.un.p.centre.pseudo.adj$padj < alpha), ]

sigtab.un.p.centre.pseudo.adj <- cbind(as(sigtab.un.p.centre.pseudo.adj, "data.frame"), as(tax_table(ps.f_un_p_sealcentre.t0.R)[rownames(sigtab.un.p.centre.pseudo.adj), ], "matrix"))

rownames(sigtab.un.p.centre.pseudo.adj) <- 1:nrow(sigtab.un.p.centre.pseudo.adj)

sigtab.un.p.centre.pseudo.adj$Species_bis <- paste(sigtab.un.p.centre.pseudo.adj$Genus,sigtab.un.p.centre.pseudo.adj$Species, sep=" ")

#write.xlsx(sigtab.un.p.centre.pseudo.adj, "sigtab.xlsx") ### do some work by hand in excel to get the names right.

sigtab.new <-read_excel("sigtab.xlsx", sheet = 1 )

levels3 <- sigtab.new %>%

group_by(Phylum, Species_bis) %>% #species_bis is a by hand in excel curated column version of Species (by default given by deseq) (to remove errors etc)

summarise(log2FoldChange = mean(log2FoldChange)) %>%

arrange(Phylum, log2FoldChange) %>%

.$Species_bis # code to create your own leveling system, in this case based on log2foldchange value and phylum

sigtab.new$Species_bis <- factor(sigtab.new$Species_bis,

levels = levels3) #implement levels in you y axis column

### plot

# (t0 VS R) pups in species level

ggplot(sigtab.new,

aes(y = Species_bis, x = log2FoldChange, color = Phylum)) +

geom_vline(xintercept = 0.0, color = "gray", size = 0.5) +

geom_point(size=3) +

theme(axis.text.x = element_text(angle = -90, hjust = 0, vjust=0.5)) +

ggtitle("Pups") +

theme_bw() +

theme(strip.background = element_rect(fill = "transparent"),

#axis.text.y = element_text(color = axis_colour),

plot.title = element_text(size = 10))+

xlab("log2FoldChange")+

ylab("Species name (if unknown higher tax. rank)")+

scale_color_manual(values = c("Actinobacteriota" = "maroon",

"Bacteroidota"="palegreen4",

"Campilobacterota"="sandybrown",

"Desulfobacterota"= "purple",

"Firmicutes"= "yellow2",

"Fusobacteriota" = "dodgerblue",

"Proteobacteria" = "lightpink"))

# removing rare species weaners

ps.f_un_w_sealcentre <- filter_taxa(ps_un_w_sealcentre, function(x) sum(x > 3) > (0.2*length(x)), TRUE)

ps.f_un_w_sealcentre

ps.f_un_w_sealcentre.pseudo <- microbiome::transform(ps.f_un_w_sealcentre, 'shift', shift = 1) # add pseudocount of 1

dds.un.w.centre.pseudo.adj <- phyloseq_to_deseq2(ps.f_un_w_sealcentre.pseudo, ~ sex + sampletype_just_for_indication) # building dds matrix

dds.un.w.centre.pseudo.adj

diagdds.un.w.centre.pseudo.adj <- DESeq(dds.un.w.centre.pseudo.adj, test = "Wald", fitType = "local")

diagdds.un.w.centre.pseudo.adj

resultsNames(diagdds.un.w.centre.pseudo.adj) #

summary(diagdds.un.w.centre.pseudo.adj) #

plotDispEsts(diagdds.un.w.centre.pseudo.adj)

# final object

res.un.w.centre.pseudo.adj <- results(diagdds.un.w.centre.pseudo.adj, contrast = c("sampletype_just_for_indication", "R", "t0"))

res.un.w.centre.pseudo.adj %>% head()

res.un.w.centre.pseudo.adj %>% summary()

alpha <- 0.01

sigtab.un.w.centre.pseudo.adj <- res.un.w.centre.pseudo.adj[which(res.un.w.centre.pseudo.adj$padj < alpha), ]

sigtab.un.w.centre.pseudo.adj <- cbind(as(sigtab.un.w.centre.pseudo.adj, "data.frame"), as(tax_table(ps.f_un_w_sealcentre)[rownames(sigtab.un.w.centre.pseudo.adj), ], "matrix"))

rownames(sigtab.un.w.centre.pseudo.adj) <- 1:nrow(sigtab.un.w.centre.pseudo.adj)

sigtab.un.w.centre.pseudo.adj$Species_bis <- paste(sigtab.un.w.centre.pseudo.adj$Genus,sigtab.un.w.centre.pseudo.adj$Species, sep=" ")

#write.xlsx(sigtab.un.w.centre.pseudo.adj, "sigtab_w.xlsx") ### do some hand work in excel to get the names right.

sigtab.new <-read_excel("sigtab_w.xlsx", sheet= 1)

levels3 <- sigtab.new %>%

group_by(Phylum, Species_bis) %>%

summarise(log2FoldChange = mean(log2FoldChange)) %>%

arrange(Phylum, log2FoldChange) %>%

.$Species_bis

sigtab.new$Species_bis <- factor(sigtab.new$Species_bis,

levels = levels3)

### plot

# (t0 VS R) weaner in species level

ggplot(sigtab.new,

aes(y = Species_bis, x = log2FoldChange, color = Phylum)) +

geom_vline(xintercept = 0.0, color = "gray", size = 0.5) +

geom_point(size=3) +

theme(axis.text.x = element_text(angle = -90, hjust = 0, vjust=0.5)) +

ggtitle("Weaners") +

theme_bw() +

theme(strip.background = element_rect(fill = "transparent"),

#axis.text.y = element_text(color = axis_colour),

plot.title = element_text(size = 10))+

xlab("log2FoldChange")+

ylab("Species name (if unknown higher tax. rank)")+

scale_color_manual(values = c("Actinobacteriota" = "maroon",

"Bacteroidota"="palegreen4",

"Campilobacterota"="sandybrown",

#"Desulfobacterota"= "dodgerblue",

"Firmicutes"= "yellow2",

"Fusobacteriota" = "dodgerblue",

"Proteobacteria" = "lightpink"))

```

SUPLLEMENTAL TABLE 2a, b, c and d

```{r}

# pups

# Observed

lme.p.obs.age <- lme(Observed ~ age_at_sampling, random =~ 1 | seal, data = df.r.un.p.alpha.div)

summary(lme.p.obs.age)

lme.p.obs.feed <- lme(Observed ~ feed, random =~ 1 | seal, data = df.r.un.p.alpha.div)

summary(lme.p.obs.feed)

lme.p.obs.sex <- lme(Observed ~ sex, random =~ 1 | seal, data = df.r.un.p.alpha.div)

summary(lme.p.obs.sex)

lme.p.obs.day <- lme(Observed ~ days_at_rehab, random =~ 1 | seal, data = df.r.un.p.alpha.div)

summary(lme.p.obs.day)

# Shannon

lme.p.shannon.age <- lme(Shannon ~ age_at_sampling, random =~ 1 | seal, data = df.r.un.p.alpha.div)

summary(lme.p.shannon.age)

lme.p.shannon.feed <- lme(Shannon ~ feed, random =~ 1 | seal, data = df.r.un.p.alpha.div)

summary(lme.p.shannon.feed)

lme.p.shannon.sex <- lme(Shannon ~ sex, random =~ 1 | seal, data = df.r.un.p.alpha.div)

summary(lme.p.shannon.sex)

lme.p.shannon.day <- lme(Shannon ~ days_at_rehab, random =~ 1 | seal, data = df.r.un.p.alpha.div)

summary(lme.p.shannon.day)

# weaners

# Observed

lme.w.obs.age <- lme(Observed ~ age_at_sampling, random =~ 1 | seal, data = df.r.un.w.alpha.div)

summary(lme.w.obs.age)

lme.w.obs.feed <- lme(Observed ~ feed, random =~ 1 | seal, data = df.r.un.w.alpha.div)

summary(lme.w.obs.feed)

lme.w.obs.sex <- lme(Observed ~ sex, random =~ 1 | seal, data = df.r.un.w.alpha.div)

summary(lme.w.obs.sex)

lme.w.obs.day <- lme(Observed ~ days_at_rehab, random =~ 1 | seal, data = df.r.un.w.alpha.div)

summary(lme.w.obs.day)

# Shannon

lme.w.shannon.age <- lme(Shannon ~ age_at_sampling, random =~ 1 | seal, data = df.r.un.w.alpha.div)

summary(lme.w.shannon.age)

lme.w.shannon.feed <- lme(Shannon ~ feed, random =~ 1 | seal, data = df.r.un.w.alpha.div)

summary(lme.w.shannon.feed)

lme.w.shannon.sex <- lme(Shannon ~ sex, random =~ 1 | seal, data = df.r.un.w.alpha.div)

summary(lme.w.shannon.sex)

lme.w.shannon.day <- lme(Shannon ~ days_at_rehab, random =~ 1 | seal, data = df.r.un.w.alpha.div)

summary(lme.w.shannon.day)

```

SUPPLEMENTAL TABLE 3

```{r}

# pups

pairwise.adonis2(ps_un_p_sealcentre.bray ~ sampletype_just_for_indication, data = df.ps_un_p_sealcentre)

# weaners

pairwise.adonis2(ps_un_w_sealcentre.bray ~ sampletype_just_for_indication, data = df.ps_un_w_sealcentre)

```
