# Supplementary material for: Characterising the gut microbiome of stranded harbour seals (Phoca vitulina) in rehabilitation
Source: PLoS One. 2023 Dec 5;18(12):e0295072. doi: 10.1371/journal.pone.0295072 (PMC10697512; doi:10.1371/journal.pone.0295072)
Supplement: Attachment — Submitted filename: Response to reviewers.docx [file pone.0295072.s011.docx]

Dear editor,

Thank you for the new opportunity to respond to the reviewers' comments on our manuscript “Characterising the gut microbiome of stranded harbour seals (*Phoca vitulina)* in rehabilitation”. We appreciate the reviewers taking once again the time to provide feedback, which has helped strengthen our work. In the revised manuscript, we have addressed all the concerns that were raised.

In the text below we hope to address all reviewers’ concerns point-by-point. We look forward to your feedback and are happy to address any other questions as you consider our revised work further.

Yours sincerely,

Ana Rubio-Garcia

PhD student, Division of Infectious Diseases and Immunology, Utrecht University Faculty of Veterinary Medicine, Utrecht, the Netherlands

Head of the Veterinary and Research Department, Sealcentre Pieterburen, Pieterburen, the Netherlands.

Reviewer #1: The revised manuscript has addressed most of the reviewers' comments, but there are still a few areas that require attention before it can be accepted for publication.

Comments:

1. The bioproject PRJEB60284 is not currently publicly available. Its availability cannot be verified at this moment. It should become publicly available before the manuscript is accepted.

**Response:** Thank you for your suggestion, in accordance the guidelines of the journal PLOS One this information will be available when the manuscript is accepted.

2. The reference format is inconsistent. The authors are advised to verify their reference formatting.

**Response:** Thank you for pointing this out, we have reviewed the reference format and adapted it to the correct one, Vancouver style.

3. When referring to figures in the text, authors are advised to use 'Fig 1a, c' instead of 'Fig 1ac.'

**Response:** We thank the reviewer for noticing this, we have adapted the references throughout the text (lines 254, 255, 266, 268 of the revised manuscript).

4. 297-299 ‘There were also significant beta diversity differences between rehabilitated and wild seals (PERMANOVA p-value<0.001, R2=0.091, and ß dispersion’s p-value = 0.002; table not shown).’ It would be beneficial to include a supplementary figure, such as a PCoA plot, to support this conclusion. This sentence should be removed if no data, figure, or table can support this conclusion.

Response: Thank you for this suggestion, we agree with the reviewer and we have added Fig S2 to support this conclusion (lines 669-673 of the revised manuscript).

Reviewer #2: Final comments:
- Line 40: add p-value where quoting 'significantly'

**Response:** Thank you for your suggestion, we have added the p values (lines 41-43 of the revised manuscript).

- Line 69-70: first sentence of paragraph feels out of place, very arbitrary here

**Response:** We thank you for pointing this out, we have adapted the text to clarify the paragraph (lines 69-70 of the revised manuscript).

- Previous comment on L154, as to why phylum level: authors' response is sufficient, but needs to be made clear in the manuscript too

**Response:** Thank you for your suggestion, we have adapted the text (lines 193-194 of the revised manuscript).

- Table 1: present means with standard deviation; also, the meaning of 'n 29 < 10' is unclear. Where does ne fit in here? does it mean n=29?

**Response:** We apologize the table was not clear, we have made adjustments to clarify the number of seals and their age and replaced the 10/90 percentile with standard deviations.

During the work on the manuscript following the reviewers feedback we came across 3 minor inconsistencies and changed the text for the better readability.

- Removed one significant association that was accidentally mentioned in the abstract and was incorrect (lines 40and 41 of the revised manuscript).
- Added to the caption of fig 2 and Fig 3 that it concerns NMDS plots (lines 274 and 281 of the revised manuscript).
- Changed the brackets around the unit ‘kg’ in table 2, 3, 4 and 5.
